# The reemerging and outbreak of genotypes 4 and 5 of Japanese encephalitis virus

**DOI:** 10.3389/fcimb.2023.1292693

**Published:** 2023-11-16

**Authors:** Weijia Zhang, Qikai Yin, Huanyu Wang, Guodong Liang

**Affiliations:** Department of Arbovirus, National Key Laboratory of Intelligent Tracking and Forecasting for Infectious Diseases, National Institute for Viral Disease Control and Prevention, Chinese Center for Disease Control and Prevention, Beijing, China

**Keywords:** Japanese encephalitis, Japanese encephalitis virus, genotypes, neglected, emerging

## Abstract

The Japanese encephalitis virus (JEV) is classified into five distinct genotypes, with genotypes 1 and 3 historically showing higher activity. These genotypes are the primary agents of viral encephalitis in the Asian continent. Genotypes 4 and 5 have remained silent in low-latitude tropical regions since their discovery. From 2009, the hidden genotype 5 suddenly emerged simultaneously in mosquitoes from the Tibetan region of China and those from South Korea in East Asia. The detection of genotype 5 of JEV in these mosquitoes was associated with cases of viral encephalitis in the local population. Similarly, in 2022, the long-silent genotype 4 of JEV emerged in Australia, resulting in a local outbreak of viral encephalitis that primarily affected adults and caused fatalities. The emergence and outbreaks of genotypes 4 and 5 of JEV present new challenges for the prevention and control of Japanese encephalitis (JE). This study not only analyzes the recent emergence of these new genotypes but also discusses their implications in the development of JE vaccines and laboratory tests for newly emerging JEV infections.

## 1 Introduction

Japanese encephalitis (JE) is a neurologic disease that is caused by infection with the Japanese encephalitis virus (JEV), which is transmitted through mosquito bites. Currently, this disease stands out as one of the most severe forms of viral encephalitis (Pierson et al., 2021). JEV is primarily spread via mosquitoes, with Culex mosquitoes (especially *Culex tritaeniorhynchus*) being the main vectors. Birds serve as natural intermediate hosts, while pigs are the amplifying hosts. Humans and mammals such as horses are the terminal hosts for the JEV (Griffiths et al., 2014; Pierson et al., 2021). The fatality rate among JE patients is as high as 20%-30%, with around 30%-50% of the survivors experiencing permanent neurological or psychological sequelae, which include aphasia, consciousness impairment, and limb paralysis (Solomon, 2006; Griffiths et al., 2014). According to World Health Organization (WHO) statistics, over three billion people living in 24 countries are at a risk of being infected by JEV. Approximately 67,900 JE cases are reported annually and these are associated with about 10,000 deaths (Campbell et al., 2011; Wang and Liang, 2015). While children have traditionally been more susceptible to JE (Halstead et al., 2023), the global widespread use of JE vaccines has led to significant reduction in pediatric cases in many regions. Interestingly, this was accompanied by a noticeable rise in adult JE cases, with some outbreaks having been noted (Wu et al., 2021; Hills et al., 2023).

**Figure 1:**
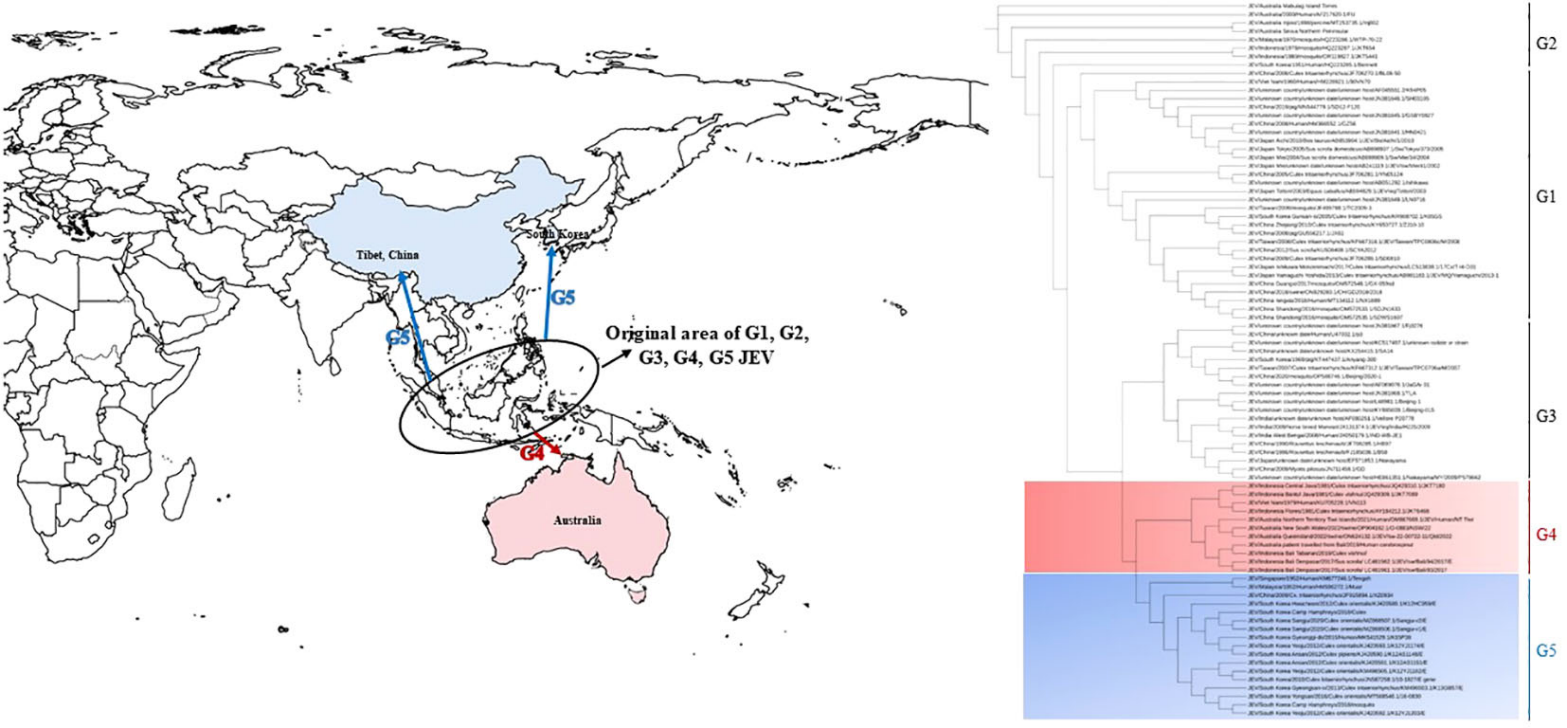
The epidemic range and phylogenetic tree of neglected genotype 4 and genotype 5 of JEV. The base layer of the modified map is sourced from Standard Map Service System, and download in EPS format from website (http://bzdt.ch.mnr.gov.cn/). The blue arrow indicates the possible transmission route of genotype 5 JEV, and the red arrow indicates the possible transmission route of genotype 4 JEV. The tree was constructed on the envelope (E) nucleotide sequences of 85 genotype1-5 JEV strains. G4 and G5 sequences are marked in light red gradient square and light blue gradient square, respectively. Genotypes are indicated on the right-side.

JEV belongs to the Flavivirus genus within the Flavividae family. It is an enveloped, single-stranded positive-sense RNA virus. The JEV genome spans approximately 11,000 nucleotides and contains a single open reading frame (ORF) that encodes three structural proteins (C, PrM/M, and E) and seven nonstructural proteins (NS1, NS2a, NS2b, NS3, NS4a, NS4b, and NS5). The viral genome is flanked by 5’ and 3’ untranslated regions (Roy, 2020; Liu et al., 2021; Pierson et al., 2021). With advancements in virus molecular biology theory and techniques, JEV has been categorized into five genotypes (G1-G5), based on the prM gene sequence (Chen et al., 1990), E gene sequence (Uchil and Satchidanandam, 2001), full genome sequence (Mohammed et al., 2011), and even the 3’ terminal untranslated region nucleotide sequence (Liu et al., 2021). Research indicates that the origin of G1-G5 is in the Indonesia-Malaysia region of Southeast Asia (**Figure 1**) (Solomon et al., 2003; Gao et al., 2013). Prior to the 1990s, genotype 3 was the predominant viral strain isolated from patients, animal hosts, and mosquitoes in JEV endemic areas. After 2000, genotypes 1 and 3 co-circulated. However, in the last decade, genotype 1 has emerged as the predominant genotype in the Asian regions that are affected by JE (Pan et al., 2011; Gao et al., 2019; Li et al., 2023).

Other genotypes of JEV have only been identified in the Indonesia-Malaysia region, particularly through long-term investigations. Genotypes 4 and 5 of JEV were not isolated from mosquitoes collected in the natural environment, animal specimens, or samples from patients with viral encephalitis, and this caused these genotypes to be neglected. However, in 2009, genotype 5 JEV was discovered in mosquitoes from the southwestern Tibetan region of China (Li et al., 2011). Subsequently, in 2010, genotype 5 was detected in mosquitoes from South Korea in East Asia. This emergence led to cases of adult viral encephalitis caused by genotype 5 JEV in the respective regions (Woo et al., 2020; Zhang et al., 2022). In 2021, Australia, which is located in the South Pacific, experienced a sudden outbreak and fatalities due to infections that were attributed to genotype 4 JEV. This marked the first occurrence of fatal viral encephalitis cases and localized outbreaks caused by genotype 4 JEV in Australia (Mackenzie et al., 2022; Sikazwe et al., 2022). The sudden emergence of genotypes 4 and 5 of JEV, along with the resulting public health burden, has not only captured the attention of virologists but also become a subject of concern to public health workers. This study provides a comprehensive review of the research progress related to the sudden appearance of genotypes 4 and 5, and their role in causing viral encephalitis.

## 2 Emerging and outbreak of genotype 4 JEV

### 2.1 Isolation and identification of genotype 4 JEV

The initial isolation of genotype 4 JEV strains dates back to between 1980 and 1981. It was restricted to mosquito specimens from regions such as Bali, Flores, and Java islands in Indonesia, and these included *C. tritaeniorhynchus* and *Culex vishnui*, among others (Schuh et al., 2013). While no reports from other areas were found, associations between the genotype 4 JEV and viral encephalitis were also unavailable. However, in 2016, the genetic sequencing of a virus strain (VN113) that was isolated from a Vietnamese patient in 1979 revealed that it was genotype 4 JEV. Although the genomic nucleotide sequence information (GenBank: KU705228.1) was published, details about the isolation and identification process, as well as patient-related information, were not disclosed. However, based on the report, this strain could be the earliest documented genotype 4 JEV.

After a span of 37 years (1980-2017) from the first mosquito-isolated genotype 4 JEV, two genotype 4 strains were isolated from domestic pig serum samples in Denpasar, Bali, Indonesia, in 2017 (JEV/sw/Bali/93/2017, GenBank: LC461961.1; JEV/sw/Bali/94/2017, GenBank: LC461962.1) (Kuwata et al., 2020). Subsequently, another genotype 4 JEV was isolated in the Tabanan Regency, only 30 km away from Denpasar, during the summer of 2019 (19CxBa-83-Cv, GenBank: LC579814.1) (Faizah et al., 2021). Notably, the distribution range of genotype 4 JEV remained extremely limited, as it only circulated among mosquitoes and animals on the Bali Island, throughout the period between 1980 and 2017.

### 2.2 Discovery of imported viral encephalitis caused by genotype 4 JEV infection in Australia

In November 2019, a 59-year-old male who was returning to Queensland, Australia from Bali, exhibited neurological infection symptoms and was later diagnosed with JE. The patient reported mosquito bites during his stay in Bali. A virus strain (Bali 2019, GenBank: MT253731.1) was isolated from his cerebrospinal fluid specimen, and it was confirmed to be genotype 4 JEV through genetic sequencing and evolutionary analysis. This marked the first confirmed case of human viral encephalitis that was caused by genotype 4 JEV (Pyke et al., 2020). The evolutionary analysis of genotype 4 JEV strains isolated from mosquitoes and patients’ samples in Bali (2017 and 2019) and those from an imported sample in Australia (2019) showed 99% nucleotide similarity. This indicated the continuous exchange genotype 4 JEV between pigs and mosquitoes in Bali, which attributed to occurrences of viral encephalitis cases (Pyke et al., 2020).

### 2.3 Emerging and outbreak of indigenous genotype 4 JEV in Australia

In February 2021, a case of viral encephalitis that resulted in the death of an adult was reported in the Tiwi Islands of northern Australia. The patient, who was a 45-year-old female, had no history of recent travel. She exhibited fever for two days, along with altered consciousness. Sadly, she passed away 15 days after onset of symptoms. Although JEV was not isolated from patient specimens, a complete JEV genome nucleotide sequence was obtained from her brain tissue (JEV/Human/NT_Tiwi Islands/2021, GenBank: OM867669.1). Evolutionary analysis of the viral genome nucleotide sequence confirmed its classification as genotype 4 JEV. This case was considered an index case for the emergence of the 2022 outbreak of the genotype 4 JEV infection in Australia (Sikazwe et al., 2022; Waller et al., 2022). Samples were collected from pig farms in Victoria, Queensland, New South Wales, and South Australia from February 25, 2022, which coincided with the local mosquito season. Subsequent testing confirmed the presence of the JEV in these samples, with positive findings in a pig pen in Victoria, six pig pens in New South Wales, and one pig pen in Queensland. Based on these findings, the Chief Veterinary Officer of Australia reported the JEV outbreak in pig farms to the World Organization for Animal Health (WOAH) on March 1, 2022 (Australian Government, Department of Agriculture, Fisheries and Forestry, 2022). Following this, JEV was also discovered in over 80 pig farms in Queensland, New South Wales, Victoria, and South Australia (Australian Government, Department of Agriculture, Fisheries and Forestry, 2023). To raise awareness and enhance preparedness for the JEV outbreak, the Acting Chief Medical Officer of Australia officially declared the event a “Communicable Disease Incident of National Significance” (CDINS) on March 4, 2022, prior to submitting case reports to the WHO (Australian Government, Department of Health and Aged Care, 2023a). The WHO subsequently shared information about the JEV outbreak in Australia to the rest of the world, urging other countries to remain vigilant by implementing necessary response measures (World Health Organization, 2022).

The occurrence of JEV in Australia persisted from January 2021 to February 2023. A total of 45 cases of JEV infection were reported across the five southeastern states of Australia, resulting in seven deaths. These five states encompass more than 50% of Australia’s land area. The prevalence of the cases was as follows: 14 cases for New South Wales; 14 cases for Victoria; 10 cases for South Australia; five cases for Queensland; and two cases for the Northern Territory (Australian Government, Department of Health and Aged Care, 2022). The total number of cases from New South Wales, Victoria, and South Australia accounted for 84% (38 out of 45) of all cases (45). The peak months for case occurrence were during the mosquito-active period from November 2021 to April 2022. Sporadic cases were also reported between November 2022 and April 2023 (Government of South Australia, 2022; New South Wales Government, Department of Health, 2022a; North Territory Government, Department of Health, 2022; Victoria Government, 2022; Queensland Government, 2023). This outbreak marked the largest indigenous JE outbreak ever recorded in Australia. According to information released by state government websites (Government of South Australia, 2022; New South Wales Government, Department of Health, 2022a; North Territory Government, Department of Health, 2022; Victoria Government, 2022; Queensland Government, 2023), a total of 21 cases had clear age information. That showed the affected people were aged between 20 to 70 years, except for three pediatric cases. Notably, there were at least eight cases (38.1%, 8/21) among individuals who were aged 60 and above. As of June 2023, no indigenous cases caused by genotype 4 JEV infection have been reported in Australia. Based on this information, Professor Paul Kelly, the Chief Medical Officer of Australia, announced that JEV infection is no longer classified as “Communicable Disease Incident of National Significance” (CDINS) in Australia on June 16, 2023 (Australian Government, Department of Health and Aged Care, 2023b).

Seroprevalence studies conducted among the population revealed that in the southern and southeastern regions of Australia, especially Victoria and New South Wales, the rates of JEV antibody positivity were 3.3% (27/820, April 2022) (Victoria Government, Department of Health, 2022), 1.4% (12/878, April 2022), and 8.7% (80/917, July 2022) (Howard-Jones et al., 2022; New South Wales Government, Department of Health, 2022b). On March 30, 2022, a case of JEV infection was identified in an alpaca in Adelaide, South Australia. This showed that JEV not only caused human outbreaks but also infected rare animals in the region. While no cases of JEV infection were found in Australia’s western region, JEV antibody positivity was detected in index chickens, and this suggested the widespread presence of genotype 4 JEV in the region (Western Australia Government, Department of Health, 2023).

## 3 Emerging and outbreak of genotype 5 JEV

### 3.1 Isolation and identification of genotype 5 JEV

Isolation from the First Human Case: In the summer of 1952, an outbreak of viral encephalitis occurred in Malaya and Singapore. A virus (referred to as Muar strain) was isolated from brain tissue specimens of a 19-year-old male patient from Malaya). This virus was identified as JEV after neutralization tests with the Nakayama strain of JEV (Hale et al., 1952; Hasegawa et al., 1994). Subsequent molecular studies revealed that the Muar strain was actually a genotype 5 JEV (Solomon et al., 2003). This was the first genotype 5 JEV to be ever identified. The case also marked the first instance of isolating genotype 5 JEV from patient tissues, thereby highlighting its potential lethality.

First Isolation from Mosquitoes: From 1952 to 2009, no further instances of genotype 5 JEV isolation were documented globally. However, a mosquito-borne virus survey conducted in 2009 in the Tibet region of China yielded a breakthrough. A virus (designated as XZ0934) was isolated from *C. tritaeniorhynchus* mosquitoes collected from local pigsties. Full genome sequencing and analysis revealed that XZ0934’s genome was 10,983 nt long, encoding an open reading frame (ORF). The phylogenetic trees constructed from sequences of structural genes (C, PrM, M, and E) consistently placed XZ0934 in the same branch as the genotype 5 Muar strain. This indicated classification of this viral strain as a new isolate of genotype 5 JEV (Li et al., 2011; Li et al., 2014). This marked the second genotype 5 JEV isolate in the world, after the initial Muar virus in 1952. It was also the first isolation of genotype 5 JEV from mosquitoes. The emergence of this virus showed its reappearance nature, even after being hidden for approximately 60 years (1951-2009), bearing notable epidemiological significance (Li et al., 2011; Li et al., 2014).

### 3.2 Genotype 5 JEV detection in mosquito vectors

In 2010, genotype 5 JEV was detected in samples of *Culex bitaeniorhynchus* mosquitoes collected in South Korea (Takhampunya et al., 2011). Subsequently, the same viral strain was detected in samples of *Culex orientalis* mosquitoes from various regions in South Korea in 2012. This brought attention to an increasing prevalence of genotype 5 JEV in mosquito populations (Kim et al., 2015). Continuous mosquito surveillance in South Korea has consistently revealed the existence of genotype 5 JEV, and the distribution of this strain seems to gradually expand enough to cover the entire country (Bae et al., 2018; Sanborn et al., 2021; Seo et al., 2021). This case marks the second discovery of the genotype 5 JEV in mosquito samples in East Asia, since its first isolation from mosquitoes in southwestern China’s Tibet region in 2009. Such reports indicated the renewed circulation of the viral strain in the natural environment. These findings underscore the reappearance and circulation of genotype 5 JEV after several decades.

### 3.3 JE cases in South Korea (2010-2015)

On August 15, 2015, a 27-year-old female patient from Gyeonggi Province, in South Korea was admitted to the hospital with symptoms that included fever, headache, apathy, and nausea. The patient had previously received JE vaccination. After hospital treatment, the patient recovered and was discharged. Despite testing negative for acute-phase JEV IgM antibodies in both the serum and cerebrospinal fluid, a genotype 5 JEV strain (K15P38) was isolated from a cerebrospinal fluid sample collected during the patient’s recovery phase using BHK-21 cell culture methods (Woo et al., 2020). This was the second instance of isolating genotype 5 JEV from patient samples, following its isolation in 1952 from a patient in Malaya.

## 4 Discussion

The re-emergence of long-hidden genotype 4 and genotype 5 JEV in Australia and South Korea, along with the disease outbreaks and accompanying fatalities among humans and animals, has raised global scientific concern. The current outbreak has largely subsided, but questions regarding whether these viral strains will reappear in Australia and South Korea remain. Some stakeholders wonder if these viruses will ever resurface in other regions across the world. These have become scientific concerns of global significance. Understanding how to predict the occurrence and spread of rare genotype JEV outbreaks in terms of timing and location has become the focal point of the discussion. While accurate prediction of viral epidemics remains challenging, analyzing the patterns of emergence observed during the re-emergence of genotype 4 and genotype 5 JEV in Australia and South Korea, along with the presence of the virus in vector and host populations, could offer valuable insights and reference points. Contemplating these phenomena can contribute to preparing for the potential re-emergence of neglected genotypes in other regions.

### 4.1 Enhanced surveillance for early detection of the JEV in nature

Published research findings indicate that both the genotype 4 JEV in Australia and the genotype 5 JEV in South Korea exhibit positive JEV gene detection in local mosquitoes prior to human cases of JE infection. In 2015, South Korea experienced cases of JE caused by genotype 5 JEV (Woo et al., 2020), marking a resurgence of this genotype after over 60 years (with the initial case having been reported in 1952 in Malaya) (Hale et al., 1952). However, genotype 5 JEV had already been detected in South Korean mosquitoes in 2010. In 2012 and 2013, genotype 5 JEV was identified in the local mosquito population (Lee et al., 2022), suggesting its presence in the natural environment before human infections were observed. Notably, the analysis of South Korean mosquitoes revealed that between 2010 and 2020, a total of 20 pools of mosquitoes tested positive for genotype 5 JEV. The pools were distributed among the mosquitoes as follows: 15 pools for *C. orientalis*; three pools for *C. bitaeniorhynchus*; one pool for *C. tritaeniorhynchus*; and one pool for *Culex pipiens*. This suggests that C. *orientalis* mosquitoes primarily serve as vectors for genotype 5 JEV transmission in South Korea, although previous investigations had identified C. *tritaeniorhynchus* mosquitoes as the primary vector for JEV (Lee et al., 2022; Shin et al., 2023). The shift in vector prevalence from *C. tritaeniorhynchus* to *C*. *orientalis* mosquitoes with regard to genotype 5 JEV transmission in South Korea is worthy of more scientific investigation. Although genotype 4 JEV led to an outbreak of JE among adults during Australia’s 2022 mosquito season, it was found that genotype 4 JEV had already been present in local mosquito samples during the 2021-2022 mosquito season that preceded the outbreak (Williams et al., 2022). The aforementioned findings suggest that the detecting and monitoring the JEV carried by mosquitoes in the natural environment are effective methods that can help to predict whether overlooked genotypic strains of JEV have the potential to cause human infections or not.

### 4.2 Raise the attention of adult JE cases

Although JE is more commonly observed in children, the resurgence of genotype 4 JEV in Australia and the emergence of genotype 5 JEV in South Korea share a common feature: the majority of the cases occurred in older age groups. In Australia, JE primarily affected individuals aged between 20 and 70 years, while in South Korea, the genotype 5 JEV infections were observed in adults, with the reported case being that of a 27-year-old patient. This emphasizes the need to detect overlooked genotypic strains of JEV in adult patients with viral encephalitis. In South Korea, the genotype 5 JEV had been circulating in the local mosquito population from 2010 to 2020, and a case of genotype 5 JEV infection in an adult patient with viral encephalitis was identified in 2015 (Woo et al., 2020). According to data from the South Korea’s Disease Control and Prevention Agency (KDCA), there has been a notable increase in adult JE cases since 2010. In 2015, South Korea reported 40 cases of JE, all of which were in individuals who were aged 20 years or older. Notably, in the region where the genotype 5 JEV case occurred (Gyeonggi Province), 11 JE cases were reported (Korea Disease Control and Prevention Agency, 2023). It’s important to determine if some of these patients were infected by the genotype 5 JEV. It’s well known that JEV infections are often subclinical, so for every reported case, there might be around 250 or more infections that go undetected (Pierson et al., 2021). Therefore, the number of the genotype 5 JEV infections in South Korea in 2015 might be significantly higher than the reported single case. In summary, focusing on JE cases, especially in adults, as well as striving to ascertain the genotypic strains of the infecting JEV can play a crucial role in preventing and controlling JE. The same applies to tracing the occurrence of overlooked genotypic strains.

### 4.3 Strengthen nucleic acid testing of samples to early identify genotypes

The WHO diagnostic criteria for JEV infection in patients includes detecting JEV IgM antibodies (by the enzyme-linked immunosorbent assay, ELISA) in acute-phase serum or cerebrospinal fluid specimens. Detecting JEV genetic positivity or isolating the virus from patient specimens are other viable options (World Health Organization, 2007). Prioritizing positive IgM antibody detection as the primary diagnostic criterion is based on the longer existence of IgM antibodies produced after a JEV infection. This makes, these antibodies easier to detect. However, IgM antibody detection cannot differentiate between different genotypes of JEV (World Health Organization, 2007). To determine the genotype of the infecting JEV, it is necessary to detect JEV nucleic acid in patient specimens and analyze viral nucleotide sequences through methods such as viral genetic sequencing. Therefore, to obtain JEV genetic sequences from patient specimens in a bid to understand the genotype, the following steps are recommended: (1) Collect early-phase serum and cerebrospinal fluid specimens since JEV viremia is short-lived (Wang et al., 2010; Zhang et al., 2011); (2) Use molecular biology methods should also be employed to detect the JEV genetic sequence in patient specimens. While laboratories often consider the presence of IgM antibodies as evidence of JEV infection, they typically do not proceed with JEV genetic testing, thereby overlooking the assessment of viral genotypes. Consequently, the understanding the specific genotype of the virus that would have infected the patient is lost, which is why molecular methods are relevant, in combination with IgM antibody testing; (3) Use a conventional PCR approach to amplify viral nucleic acids, followed by sequencing to obtain the nucleotide sequence. Real-time Quantitative PCR (RT-qPCR) is widely used for viral nucleic acid detection due to its speed and efficiency. However, this method only provides qualitative detection and cannot determine viral genotypic variations; (4) Specimens from the recovery phase of the illness can also be utilized for viral isolation. It is commonly believed that the acute-phase specimens (serum/cerebrospinal fluid) of patients are the optimal choice for viral isolation. However, the isolation of the genotype 5 JEV strain in South Korea in 2015 was derived from a patient’s recovery-phase cerebrospinal fluid specimen; (5) Considering that using the method of detecting IgM antibodies for JEV infection is challenging when dealing with cases caused by genotype 4 and genotype 5 JEV, including JEV nucleic acid testing as a routine pathogen detection procedure for patients with viral encephalitis during mosquito season is recommended. This measure aims to enhance the identification of patients who are infected with genotype 4 and genotype 5 JEV.

### 4.4 Develop new JE vaccines

JE is a vaccine-preventable disease. The current JE vaccines, be they inactivated vaccines (Nakayama, P3, Beijing-1) or live attenuated vaccines (SA14-14-2), all use genotype 3 JEV as the vaccine strain (World Health Organization, 2014). However, research results indicate that the current vaccines provide full protection against genotype 3 JEV infection. However, the protection rate against genotype 1 JEV infection ranges between 92% and 97% (Fan et al., 2012; Erra et al., 2013). Other studies showed that effective protection against genotype 5 JEV infection is achieved when the neutralizing antibody titer in the human body reaches 1:320 (Cao et al., 2016). It is known that achieving neutralizing antibody titers of 1:320 through JE vaccination is a challenge. In 1982, South Korea included the JE vaccine in its immunization program, leading to a positive rate (98.1%) of neutralizing antibodies against JEV in the general population (Lee et al., 2016). In 2015, a case of a patient who had been vaccinated against JE but still contracted genotype 5 JEV was reported, despite the relatively high level of population immunity to JEV in South Korea. This highlights the urgent need to develop vaccines targeted JEV genotypes other than genotype 3. Alternatively, a recombinant vaccine that provides full coverage against infections by all five genotypes of JEV could be developed.

### 4.5 Strengthen the monitoring of JEV gene changes

As mentioned earlier, JEV originated in the Indonesia-Malaysia region and can be classified into five genotypes. Before the year 2000, genotype 3 virus was the predominant genotype of JEV. Currently, genotype 1 virus has become the dominant genotype. In recent years, the emergence of genotype 4 and genotype 5 JEV in Australia and South Korea, raises questions about potential changes in the geographic distribution of JEV genotypes. Does this signify a new shift in the predominant genotype of the Japanese encephalitis virus (Gao et al., 2019)? To address these questions, it is essential to enhance methods for detecting and monitoring JEV genotypes in the natural environment. Furthermore, while East Asia and Southeast Asia are traditional endemic areas for JE, the virus has expanded its range beyond these regions. In Italy, which is located in Europe, mosquitoes and birds tested positive for genotype 3 JEV RNA (Ravanini et al., 2012; Preziuso et al., 2018). In 2015, Angola, which is in Africa, reported its first indigenous case of genotype 3 JEV infection, marking the first case within a non-endemic region for JE (Simon-Loriere et al., 2017). These findings indicate that JEV has appeared in the natural environment of regions outside its traditional endemic areas, including Africa and Europe. The JEV continues to spread, thereby further emphasizing the need to predict its trends and range of expansion.

## Author contributions

WZ: Conceptualization, Methodology, Writing – original draft. QY: Software, Validation, Writing – original draft. HW: Project administration, Validation, Writing – review & editing. GL: Conceptualization, Formal Analysis, Supervision, Writing – review & editing.
