# Supplementary material for: The reemerging and outbreak of genotypes 4 and 5 of Japanese encephalitis virus
Source: Front Cell Infect Microbiol. 2023 Nov 16;13:1292693. doi: 10.3389/fcimb.2023.1292693 (PMC10698470; doi:10.3389/fcimb.2023.1292693)
Supplement: Supplementary file 1 [file DataSheet_1.docx]

Supplementary Material

The emerging and outbreak of genotypes 4 and 5 of Japanese encephalitis virus

Weijia Zhang, Qikai Yin, Huanyu Wang*, Guodong Liang*

*** Correspondence:** Corresponding Author: Huanyu Wang wanghy@ivdc.chinacdc.cn
 Guodong Liang gdliang@hotmail.com

# Supplementary Data

Study Procedure

The data of this study was derived from the NCBI and government websites. According to the search rules of PubMed, the search condition of genotype 5 JEV was set to: (Japanese encephalitis virus) AND ((genotype 5) OR (G V) OR (genotype V)). The search condition of genotype 4 JEV was set to: (Japanese encephalitis virus) AND ((genotype 4) OR (G Ⅳ) OR (genotype Ⅳ)). Next, we selected the articles that meet our requirements from the search results, and read the references at the end of the article to check and fill in the relevant literature content. Besides, we looked up the epidemiological information about JE on the website of the national health administrations. Then we systematically analyzed articles and website content to describe the changes in the geographical distribution of genotype 4 and genotype 5 JEV, respectively, and the extent and scale of viral encephalitis.

For the search for JEV sequences, we inputted a genotype 4 or genotype 5 JEV sequence as a query sequence into the NCBI website, used Basic Local Alignment Search Tool to blast out and downloaded all other JEV sequences of the same genotype. Sequences of genotype 1, genotype 2, and genotype 3 JEV were obtained in the same way, but only sequences representative of the strains were selected for evolutionary analysis. Molecular phylogenetic analysis was conducted using all the sequences described above.

A simplified illustration of the data collection procedure associated with our review is presented in the following road-map:

Search articles from PubMed

Search relevant information from government websites

Select articles meet our requirements

Read references of other articles to check and fill relevant literature

Systematically analyzed articles and website

JEV sequences obtained by the BLAST tool on the NCBI website

Molecular phylogenetic analysis
